# Development of an automated fruit classification system by using computer vision and deep learning

**DOI:** 10.1016/j.ohx.2026.e00775

**Published:** 2026-04-20

**Authors:** Thi-Thoa Mac, Huy-Anh Bui, Duc-Vinh Pham, Hoang-Hiep Ly, Xuan-Thuan Nguyen

**Affiliations:** a School of Mechanical Engineering, Hanoi University of Science and Technology, No 1 Dai Co Viet, Hanoi, Viet Nam; b School of Mechanical and Automotive Engineering, Hanoi University of Industry, No. 298 Cau Dien Street, Hanoi, Viet Nam

**Keywords:** Arduino, PLC, object detection, computer vision, deep learning, YOLOv8

## Abstract

With the rapid development of technology in the Industry 4.0 era, computer vision and deep learning have emerged as key technologies supporting industrial systems such as classification and quality inspection with high accuracy, optimizing processes in production lines. In recent years, computer vision and data-driven intelligent systems have played an increasingly important role in industrial automation. Moreover, smart warehouse systems minimize unnecessary steps in product storage, optimizing the time required for import and export in industrial production lines. This paper proposes an automated import/export system integrated with the YOLOv8 network to classify various fruits (kumquats, longans, cherry tomatoes) and store them in an automated warehouse system. The import/export data is recorded on a personal web platform to track the system’s input and output volumes. A multi-threading mechanism is also applied to ensure real-time data processing. Experimental results indicate that the overall system achieves a high accuracy rate of approximately 98%. A demonstration video illustrating the system operation and setup procedure is available in the project repository.

## Graphical abstract

**Figure:**
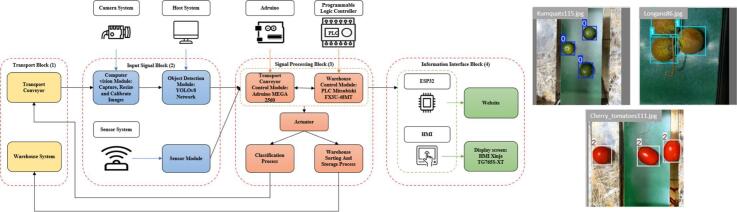

**Specifications table**

**Table:** 

Hardware name	*Automated Fruit Classification System by Using Computer Vision and Deep Learning*
Subject area	• Engineering and materials science • General
Hardware type	• Imaging tools • Measuring physical properties and in-lab sensors • Electrical engineering and computer science • Mechanical engineering and materials science
Closest commercial analog	No commercial analog is available
Open Source License	CC BY-NC 4.0 (Hardware)AGPL-3.0 license (Software)
Cost of Hardware	1500$
Source File Repository	https://doi.org/10.17632/dtt62jncwh.2

## 1 Hardware in context

These days, the use of computer vision alongside intelligent data-driven systems is playing a big role in how tasks are automated. It allows machines to handle detailed operations more quickly and accurately than manual methods. This is a reason why the traditional industries have seen real benefits and improvements from applying machine vision. Computer vision gives machines a kind of “sight”, letting them interpret images and video in ways that help them react to their surroundings. By learning from patterns in the data, machines can gradually get better at recognizing what they see and make more suitable decisions as they go [1]. In real-world settings, this has made a big difference. For example, in manufacturing, image-based systems can spot tiny defects that human inspectors might miss [2]. In robotics, vision systems help machines move through complex spaces, find objects, and perform tasks like assembling or fixing equipment with greater accuracy. What's making this possible is a method called deep learning, which lets systems process huge amounts of visual data to figure out what's in an image. Tools such as the YOLO network are designed for high-speed inference and can perform real-time object detection, making them well suited for applications such as product sorting, defect inspection, and security monitoring [3]. Because these systems can respond to what’s happening around them in real time, they’re becoming essential in modern production setups. One of the latest versions of this tech, called YOLOv8, has shown it can identify items quickly and precisely, which is a big help in automated systems for shipping and logistics [4]. By accelerating the processing speed and improving identification accuracy, the YOLOv8 model enables warehouse management systems to operate in real time [5]. Likewise, because of its stability under continuous running settings [6], YOLOv8 improves the dependability of automated commodities processing and categorization systems. Moreover, using YOLOv8 reduces mistakes in inventory control and categorization of products, therefore satisfying the need for efficiency and speed in systems of continuous operation [7]. The YOLOv8 model in automated systems represents significant progress in artificial intelligence object recognition applications. When enhanced with spatial pyramid pooling and route aggregation networks, YOLOv8 known for its exceptional accuracy and quick inference speed—is well-suited for real-time applications in challenging industrial and agricultural situations. Studies have shown that the model can identify tiny and densely packed objects, essential for automated systems needing to manage heavy workloads and provide dependable detection under diverse environments. For jobs like inventory monitoring or automated quality control on manufacturing lines, YOLOv8 has improved feature flow via its PANet structure, guaranteeing effective detection over many scales [8]. Recently, many studies on computer vision, artificial intelligence models, and logic controller development have been reported. With particular focus on the removal of metallic pollutants, Dhaval Tailor et al. provided the design of a reasonably cost automation system meant for sorting and stacking lightweight objects. It underlines especially how industrial sectors are focusing more and more on automation to increase output and reduce human error as product demand increases. Combining sensors and motors with a programmable logic controller (PLC) to track the sorting process, the system precisely recognizes and sorts objects without human interaction [9]. Analyzing current approaches and advancements in deep learning-based object recognition, Xiongwei Wu et al. presented an understanding of deep learning networks and support the development of more complex applications in sectors including autonomous vehicles, security surveillance, and industrial automation systems [10]. Using multi-view deep learning techniques for object identification to reduce occlusion and blind spots, Cong Tang et al. overcame typical challenges in real-time applications [11]. Li Liu et al. investigated the local challenges and gave a comprehensive overview of advancements in object recognition using deep learning techniques [12]. Nils Hütten et al. investigated how deep learning may be used in automated visual inspection as it helped to uncover flaws in manufacturing and maintenance processes and reduced the need for human inspections [13]. Using the Universal Robotics UR3e robotic arm, which ran the Raspberry Pi 5 module and PiCamera 3 to assess fruit quality in real-time, Pedro Dinis Gaspar reported on the creation of a low-cost system allowing automatic identification and processing [14]. The work developed four item recognition models using TensorFlow's object identification API with notable fruit classification accuracy. The method guaranteed constant product standards, therefore enabling non-destructive fruit inspection and hence minimizing waste by improving quality control. In the end, it raised product quality and consumer pleasure. Erik Kucera et al. underlined how computer vision and machine learning might help increase accuracy in industrial operations [15]. These technologies were shown to enhance production and quality control. Furthermore, the article discussed the possibilities of these technologies to solve problems in many fields, thereby opening the path for better operational results and smart manufacturing ideas. Emphasizing Low-Shot Object Detection (LSOD), an area comprising approaches including One-Shot Object Detection (OSOD), Few-Shot Object Detection (FSOD), and Zero-Shot Object Detection (ZSOD), Qihan Huang et al. provided a comprehensive review of techniques for spotting objects in scenarios where labeled data was rare [16]. Qingwang Wang et al. proposed a deep learning-based technique for defect identification in chip packing and reached an accuracy of up to 93.3% in real-world circumstances [17]. Specifically focused on robotic grab detection applications employing a two-finger gripper and an RGB-D camera combined with a collaborative robot, Yassine Yazid et al. addressed developments in robotic vision. They underlined how edge computing units and a visual recognition system were integrated to allow picture recognition of damaged objects [18]. This procedure defined defect outlines, detected deformities in object pictures using a modified version of the You Only Look Once (YOLO) technique, and told the robot controller to remove malfunctioning components. On customized data, the gadget performed remarkably with a 96.6% grip accuracy. It also achieved a 96% detection accuracy. The research illustrated the efficiency of the YOLOv5 algorithm in industrial applications. It emphasized the algorithm's balance between accuracy and recall. With special focus on Convolutional Neural Networks (CNNs) and their integration with hardware accelerators for industrial use, Muhammad Hussain et al. provide computer vision (CV) architectures [19]. The study stressed the importance of appropriate datasets to increase CV applications' performance benchmarks and the criteria for creating flexible hardware solutions.

## 2 Hardware description

This project comes from an idea about how deep learning can help with computer vision and automation. It aims to build a system that can sort fruits automatically. To do this, it uses a modern image recognition model known as YOLOv8. Key contributions of the paper are as follows:

• The whole system is designed using deep learning methods from the ground up.
• The communication between control units and motors is set up to work smoothly and without delay.
• A basic online website is added to let users’ access and use the system more easily.
• Different versions of YOLOv8 are tested to show how well deep learning performs in recognizing and classifying three kinds of fruit, which are the main focus of this study.

### 2.1 The Proposed System

**Fig. 1:**
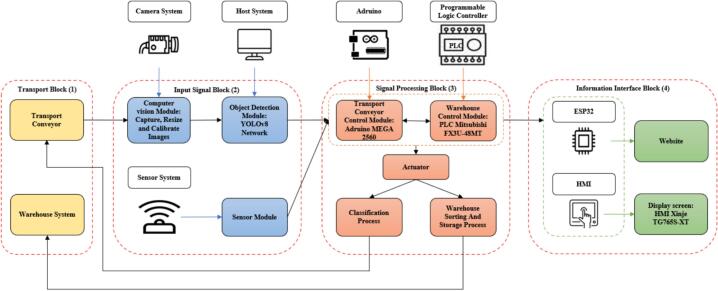
The main structure of the proposed system.

The overall structure of the proposed system is indicated in Fig. 1. To be more detailed, the proposed system consists of four main blocks: the transport block (1), input signal block (2), signal processing block (3), and information interface block (4). The transport block (1) includes the transport conveyor and the warehouse system. This block performs two main functions. The first one is moving the unclassified samples to the input signal block (2). The second one is dividing the detected samples into appropriate warehouse areas. The input signal block (2) contains three sub-modules: a computer vision module, an object detection module, and a sensor system module. This block utilizes the signal data from the sensors and camera system to identify and capture images of the fruit samples. The captured images are then used to determine types of fruit. Thereafter, the signal processing block (3) uses this information to control actuators for the classification process as well as the warehouse storing operation. The Arduino microcontroller processes the data from the transmission line on the host system and creates demands to ensure each sample is delivered to the proper sorting zones. Additionally, the smart PLC-based warehouse system automatically moves the storage boxes into the appropriate compartments after the number of fruits reaches the limit. The system operates these two submodules in parallel to maintain real-time speeds. The information interface block (4) comprises a monitor screen to display the classified results and a host website for user interaction. This website is also executed online to keep track consistently of the quantities as well as the prices for each kind of product.

**Fig. 2:**
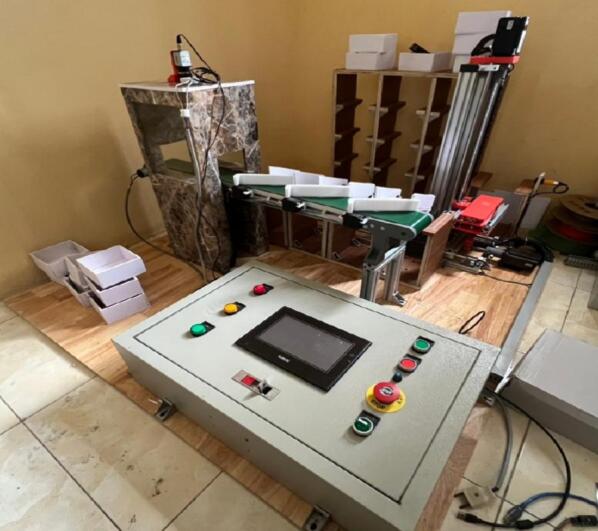
The proposed system in reality.

Fig. 2 shows the completed mechanical system after finishing the manufacturing process. While the essential components remain consistent with the 3D design, we have added some minor modifications related to the material selection. As shown in Fig. 2, we primarily utilize T-slot aluminum profiles for the storage, which reduces weight and maintains the structure's strength. Overall, the consistency between the design and the implemented version demonstrated the transition from conceptualization to a working prototype as well as revealed the feasibility of the proposed system.

### 2.2 The YOLOv8 network

**Fig. 3:**
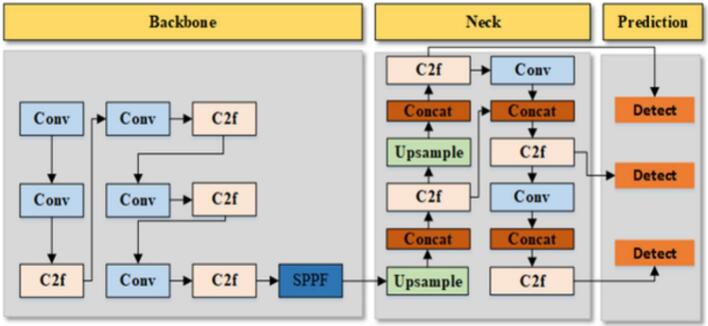
The structure of the YOLOv8 model [8].

The YOLOv8-based deep learning model is utilized for automatic fruit identification and classification due to its high detection accuracy and fast inference capability. It inherits many advantages of previous versions in the YOLO family. Fig. 3 shows the detailed structure of the standard YOLOv8 network. There are three main parts inside the model: The Backbone, the Neck, and the Head (also called the Prediction).

#### 2.2.1 The structure of the model network

The Backbone of YOLOv8 is the object recognition process, which is based on the extraction of salient information from the input image. The Backbone is used to identify edges, textures, and shapes among other basic to complex components. Inspired by CSPNet, one interesting component meant to improve Backbone processing performance is a Cross-Stage Partial (C2f) network. This method reduces the necessary number of parameters, thus preserving important feature information and consequently improving processing capacity. By use of the Spatial Pyramid Pooling Fast (SPPF) layer, the Backbone additionally combines data from many scales, therefore allowing the model to detect objects of different sizes. The Backbone of YOLOv8 uses a step-by-step structure from stages P1 to P5 to gather all the important details before the model processes them further.

The Neck of YOLOv8 is an intermediary part between the Backbone and the Head. The Neck is designed to optimize the combination of information from multiple feature levels. By using a mechanism that integrated the Upsample layers (for size increasing) and the Concat layers (for feature combining), the Neck of YOLOv8 can maximize the amount of data from both high- and low-resolution layers. Since then, the Neck has provided the model with a comprehensive view of objects in the image. This enables the model to maintain accuracy for larger objects while enhancing detection performance for intricate objects. In other words, the Neck is a crucial component responsible for synthesizing and fine-tuning the extracted features.

The Head of YOLOv8 is the third part of the model. To be more detailed, the Head uses an anchor-free structure to simplify the computation process. By using both the convolutional layers and customized output layers, the Head of YOLOv8 directly predicts the centroids of targets and bounding box sizes for each feature level. As another word implies, the head predicts the shape and position of the image's objects, along with the feature layers of the Neck. Moreover, the Head also provides robust object detection capabilities for the model, especially in complex situations involving multiple objects in the same image frame.

#### 2.2.2 The Loss Function

The loss function in YOLOv8 is used to measure and optimize the accuracy of object detection. Hence, it is designed to balance three main components: the Bounding Box Regression Loss ($L_{\mathrm{Box}}$), the Classification Loss ($L_{\mathrm{Cls}}$), and the Distribution Focal Loss (DFL) ($L_{\mathrm{DFL}}$).The general loss function of YOLOv8 is expressed as:

(1) $$L_{YOLOv8}=\varpi_{\mathrm{Box}}.L_{\mathrm{Box}}+\varpi_{\mathrm{Cls}}.L_{\mathrm{Cls}}+\varpi_{\mathrm{DFL}}.L_{\mathrm{DFL}}$$

whereas the parameters $\varpi_{\mathrm{Box}}$,$\varpi_{\mathrm{Cls}}$,$\varpi_{\mathrm{DFL}}$ are weight adjustment coefficients for each loss function. They are established to figure out the suitable influence of each type of loss function during the optimization process.

To be more detailed, the Bounding Box Regression Loss evaluates the accuracy of the model in predicting the coordinates of the bounding boxes around objects. This loss function aims to minimize the difference between the predicted and actual positions, sizes, and shapes of the boxes. By using the Complete Intersection over Union (CIoU) metric, the Bounding Box Regression Loss is computed as:

(2) $$L_{\mathrm{Box}}=1-CIoU$$

Equation (3) presents the function of the *CIoU* metric:

(3) $$\mathrm{CIoU}=\left(1-IoU\right)+\frac{d^{2}\left(B^{b},B^{g}\right)}{d_{\mathrm{diagonal}}^{2}}+\alpha v$$

where: *IoU* denotes the ratio of the overlapping area between the predicted bounding box ($B^{b}$) and the ground truth ($B^{g}$) relative to the total area of both regions.

δ is the Euclidean distance between the center of ($B^{b}$) and ($B^{g}$).

$d_{\mathrm{diagonal}}$ is the diagonal distance of the bounding box that surrounds both ($B^{b}$) and ($B^{g}$).

α and v are the parameters that adjust the difference in aspect ratio between ($B^{b}$) and ($B^{g}$).

The Classification Loss focuses on the model's ability to assign the correct class labels to the detected objects. By utilizing the Binary Cross-Entropy (BCE) function, the formulation of the Classification Loss is written as follows:

(4) $$L_{\mathrm{Cls}}=-\frac{1}{M}\sum_{i=1}^{M}\sum_{c=1}^{C}[y_{\mathrm{ic}}\times \log\left({\hat{y}}_{\mathrm{ic}}\right)]$$

where: *M* denotes the number of object samples in the image and *C* denotes the number of classes.

$y_{\mathrm{ic}}$ and ${\hat{y}}_{\mathrm{ic}}$ are the actual label and predicted probability for class *c* of object *i*, respectively.

The negative sign in Equation (4) converts the log-likelihood maximization problem into a minimization objective, which is consistent with gradient-based optimization frameworks used in deep learning. The term $\frac{1}{M}$ serves as a normalization factor over the number of sampled objects in the batch, where M denotes the number of sampled objects. The negative sign does not influence the sampling strategy, the number of objects M, or the classification behavior itself. It is used to ensure that minimizing the loss corresponds to maximizing the likelihood of correct predictions during training.

The Distribution Focal Loss (DFL) is designed to optimize the accuracy of the bounding box, particularly in complicated situations, such as when dealing with small objects or slight position differences. The formulation of the Distribution Focal Loss is presented as:

(5) $$L_{\mathrm{DFL}}=\sum_{i=1}^{N}\mathrm{softmax}\left(d_{A}\right).\text{log}\left(true\_dist_{A}\right)$$

where: $d_{A}$ represents the predicted value of the bounding box at the coordinate “*A”*, $true\_dist_{A}$ represents the true distribution of the appropriate predicted values, $\mathrm{softmax}(d_{A})$ is the SoftMax function used to normalize the predicted values into a probability distribution.

As shown in Equation (5), the DFL function is formulated as a cross-entropy loss over discretized bounding box offset distributions. Since cross-entropy is not upper-bounded, its value is not constrained within the range [0,1]. The reported DFL value corresponds to the averaged validation loss over positive samples and is not a normalized probability metric. Furthermore, the DFL component is incorporated into the overall YOLOv8 loss formulation with a predefined weighting coefficient during training.

Potential applications of the proposed hardware system include:

• Enabling automated fruit sorting for small-scale agricultural processing systems.
• Providing a modular platform for integrating computer vision with industrial control systems.
• Serving as a laboratory testbed for studying real-time embedded system communication.
• Supporting the prototyping of smart warehouse and inventory management solutions.
• Allowing researchers to investigate scalable sorting mechanisms.

## 3 Design files summary

**Fig. 4:**
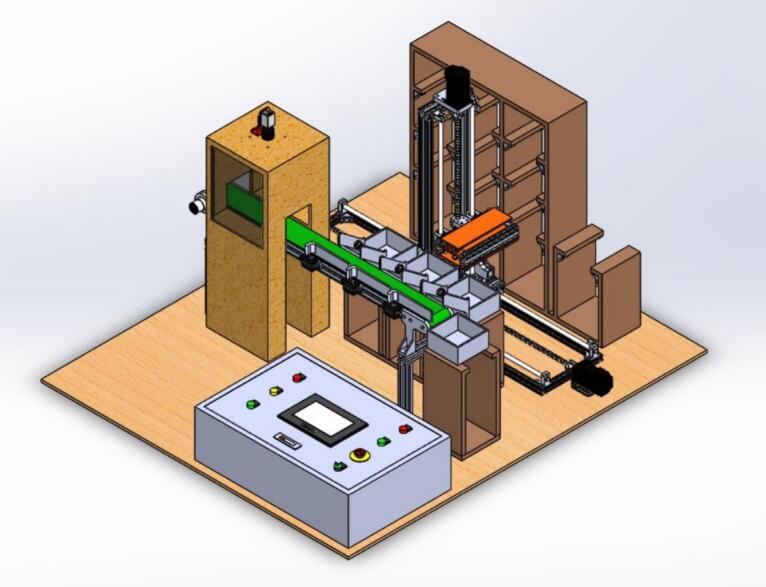
Overview of system 3D design.

The overall 3D system design is clearly illustrated in Fig. 4. To fully understand the components included in the system, access the CAD_ASAR files to directly review the sectional drawings and the 3D files to view the mechanical design files for each system's parts. The code for accessing the camera and the image processing system, which applies the trained model (the trained model is the best.pt file and the dataset used for training is trains.zip), is implemented in the Detectyolov8.py file. Regarding the control system and the display interface, the system is divided into two control processing parts: the classification section and the smart sorting section. These correspond to the Code_mega.ino file for the classification control processing and the PLC.gxw file for the smart sorting control processing. For the display interface, the HMI.txp file builds the overall interface for system visualization and operational monitoring, while the ESP32_code.ino and web.zip files are responsible for pushing parameters and building the web-based system. The system design files which correspond to their access paths are shown in Table 1.

**Table 1:** Design files of the system.

Design file name	File type	Open source license	Location of the file
CAD_ASRS	.dwg	CC-BY 4.0	https://doi.org/10.17632/dtt62jncwh.2
3D	.zip	CC-BY 4.0	
PLC	.gxw	CC-BY 4.0	
HMI	.txp	CC-BY 4.0	
RS232	.ino	CC-BY 4.0	
Code_mega	.ino	CC-BY 4.0	
ESP32_code	.ino	CC-BY 4.0	
Detectyolov8	.py	CC-BY 4.0	
Best	.pt	CC-BY 4.0	
trains	.zip	CC-BY 4.0	
web	.zip	CC-BY 4.0	

## 4 Bill of materials summary

The prices of the devices are clearly detailed in Table 2, Table 3, Table 4, corresponding to the stages of system operation. It is noted that some parts, which were custom-designed and created via 3D printing, are not listed with a price in the tables.

**Table 2:** The bill of classification system.

**Designator**	**Component**	**Number**	**Cost per unitUSD**	**Total costUSD**	**Source of materials**	**Material type**
Motor	JGB37-520 24VDC Gear Motor	1	50$	50$	Amazon.com: JGB37-520 DC 6V/12V/24V High Gear Motor All Metal Gear Box Electric Motor 7/12/22/35/45/66/107/200/320/600/960RPM (Size : 107rpm, Color : 12V) : Industrial & Scientific	Steel, aluminum alloy, ABS plastic (gearbox housing), copper (winding)
Servo	MG996R Servo Motor	3	7$	21$	Amazon.com: 6pcs MG995 55G Micro Servo Motor Metal Geared Motor Kit for RC Car Robot Helicopter, Mini Servos for Arduino Project : Toys & Games	Nylon (housing), metal gears (steel and copper alloy), copper (wiring), electronic circuit
Speed controller	DC Motor PWM Speed Controller Module DC-DC 4.5V-35V 5A 90W	1	8$	8$	4PCS 5A 90W PWM DC Motor Speed Controller, Adjustable DC 4.5V-35V Driver Motor Speed Regulator Built-in LED Indicator with Speed Control Knob - Amazon.com	FR4 (PCB board), electronic components (semiconductors, resistors, capacitors), plastic knobs
Adapter	5V 5A Adapter	1	10$	10$	DIGISHUO DC 5V 5A 25W Power Supply Adapter Transformer Switch AC 110V/220V to DC 5V 260amp Switching Converter LED Driver for LED Strip Light CCTV Camera Security System - Amazon.com	Aluminum alloy (housing), FR4 (PCB board), electronic components (transformer, capacitors, semiconductors)
Camera	Dahua A52-01CG50E Camera	1	350-500$*	350-500$	Dahua MV-A5201M/CG50E Price Datasheet Area Scan Cameras	Aluminum alloy (housing), glass (lens), electronic components (sensors, PCB)
Controller	Arduino R3 Uno	1	9$	9$	Amazon.com: hiBCTR arduino uno r3 Board with USB Cable - atmega328p, 16mhz, 14 Digital i/o pins, pwm Support, Compatible with arduino uno r4, mega, Leonardo and Raspberry pi : Electronics	FR4 (PCB board), electronic components (microcontroller, connectors, capacitors, resistors), plastic headers

*The Dahua MV-A5201M/CG50E industrial camera is typically sold through industrial distributors. The market price varies depending on supplier availability and lens configuration. The typical price range is approximately USD 350–500 based on distributor quotations.

**Table 3:** The bill of smart sorting system.

**Designator**	**Component**	**Number**	**Cost per unitUSD**	**Total costUSD**	**Source of materials**	**Material type**
Motor	57J1880EC-1000 Gear Motor with Encoder + JMC 2HSS57 Stepper Motor Driver	2	54$	108$	Wholesale 57J1880EC-1000-LS+2HSS57 Nema 23 Hybrid Closed Loop Stepper Motor Driver for CNC Textile Engraving Machine	Steel (motor shaft, housing), aluminum alloy (housing, gearbox), copper (wiring), plastic (encoder housing, insulation parts)
	NEMA 17 Stepper Motor (42 Stepper Motor)	1	8$	8$	Twotrees Nema 17 Stepper Motor 5PCS 17HS4401 NEMA17 42 Motor 4-Lead 42	Steel (housing, shaft), aluminum alloy (end caps), copper (wiring), plastic (insulation parts)
	TB6600 Stepper Motor Driver Module	1	12$	12$	Twotrees TB6600 Stepper Motor Driver Nema 23 Nema17 4A DC9-42V for NEM	Aluminum alloy (housing/heat sink), FR4 (PCB board), electronic components (ICs, capacitors, connectors), plastic (connectors, insulation parts)
Adapter	Mean Well 24V 14.6A Power Adapter	1	42$	42$	MEAN WELL LRS-350-24 350.4W 24V 14.6A Single Output Vietnam | Ubuy	Aluminum alloy (housing), FR4 (PCB board), electronic components (transformers, capacitors, semiconductors, plastic (connectors and insulation parts)
Controller	Mitsubishi PLC FX3U-48MT/ES	1	340$	340$	Mitsubishi FX3U-48MT/ES-A – DDPARTS SOLUTION	ABS plastic (housing), FR4 (PCB board), electronic components (microcontrollers, capacitors, semiconductors), copper (connectors)
Sensor	E3F-DS30C4 Photoelectric Sensor	4	9$	36$	electrical center b2c | E3F-DS30C4 Proximity Switch Photoelectric sensor switch NPN PNP	ABS plastic (housing), glass (lens), copper (wiring), electronic components (sensors, PCB)
Limit switch	D35 V-156-1C25 Limit Switch	6	4$	24$	V-156-1C25 Microswitch/Miniature Switch by OMRON	ABS plastic (housing), metal alloy (lever), copper (internal contacts), stainless steel (spring/lever)
Button	LA38-11BN Push Button Switch	3	3$	6$	LA38-11BN Jog Power Button Self-Locking Self-Reset Switch Red And Green Hole 22m | eBay	ABS plastic (housing and button cap), copper (internal contacts), stainless steel (spring)
	LA38-11ZS Emergency Stop Button Switch	1	9$	9$	LA38-11ZS Red/Green 22mm Emergency Stop Push Button Switch Mushroom Head 10A | eBay	ABS plastic (housing and button cap), copper (internal contacts)
Light	AD16-16C 24VDC Power Light	3	1$	3$	10pc Pilot light Orange Led Lamp φ16mm Screw Type ACDC 24V Shinohawa AD16-16D/S | eBay	ABS plastic (housing and lens), LED (light source), copper (internal contacts)
Aptomat	Delixi RCBO	1	3.5$	3.5$	Delixi Dz47pley-63 63A 230AC 1p+N 50Hz“ Phase Line + Neutral Line” Residual Current Operated Circuit Breaker - Earth Leakage Circuit Breaker and Air Circuit Breaker	ABS plastic (housing), copper (internal contacts), steel (springs and terminals), ceramic (arc chamber, insulation parts)

**Table 4:** The bill of control and interface system.

**Designator**	**Component**	**Number**	**Cost per unitUSD**	**Total costUSD**	**Source of materials**	**Material type**
Controller	ESP32 Board	1	35$	35$	ESP32-DevKitC-32E Development Board Module CP2102 Driver ESP-32 DevKitC ESP-32E | eBay	FR4 (PCB board), electronic components (microcontroller, WiFi/Bluetooth module, capacitors, resistors), plastic (headers)
	Arduino Mega 2560	1	13$	13$	ATMEGA 2560 CH340 R3 Board Atmega2560-16AU Mega2560 R3 Compatible for Arduino | eBay	FR4 (PCB board), electronic components (microcontroller, connectors, capacitors, resistors), plastic headers
HMI	Xinje TG765S-XT HMI Screen	1	160$	160$	XINJE TouchWin Model TG765S-XT Touch Panel DC24V 4W Used With Warranty See Pics | eBay	ABS plastic (housing), glass (touchscreen/display), electronic components (PCB, ICs), aluminum alloy (mounting frame)
Module	RS232 to TTL Converter Module	1	2$	2$	MAX3232 RS232 Serial Port To TTL Converter Module Female DB9 COM Serial MAX2hm | eBay	FR4 (PCB board), electronic components (MAX232 or SP3232 chip, capacitors), plastic (connectors, header pins), copper (traces and pins)

## 5 Build instructions

Required tools: hex key set, screwdriver, adjustable wrench, soldering iron, and multimeter.

Regarding the build instructions, the main structure of the system is built based on three sections: Classification section, Smart Sorting section and the Interface section visualizes the data and the operational workflow of the system's various sections.

### 5.1 Mechanical construction

#### 5.1.1 Classification section

**Fig. 5:**
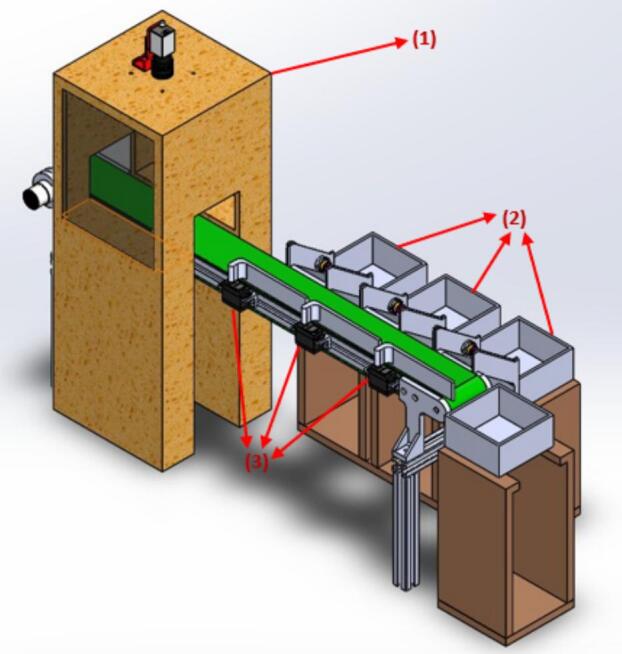
The construction architecture of the classification section.

To ensure the operating principle and safety conditions, the classification model's components are calculated and selected to meet durability requirements and function effectively in the working environment. The system includes a camera housing for object recognition (1), three boxes for categorized objects (2), and three servos to perform the sorting (3). The classification section was constructed and assembled as shown in Fig. 5.

#### 5.1.2 Smart sorting section

**Fig. 6:**
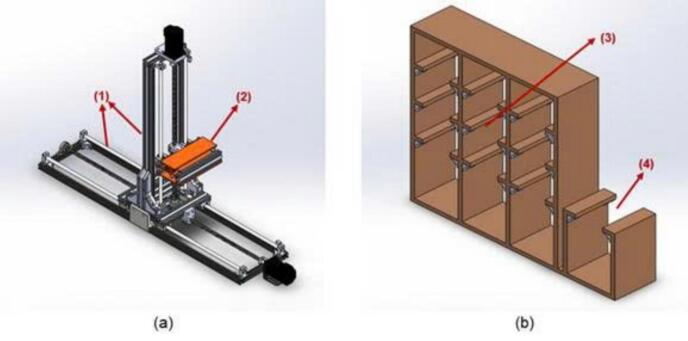
Mechanical architecture of the smart sorting subsystem: (a) transfer mechanism, (b) storage

To accommodate the incoming products with dimensions (*L* x *W* x *H*) of 12cm × 12cm × 6cm and a weight of 200g, packaged in a box containing 6 products (kumquats, longans, cherry tomatoes), and where the handling arm moves along three axes - X (running along the warehouse length), Y (moving products into and out of the warehouse), and Z (raising and lowering products within the warehouse) - the smart storage model is designed and calculated to ensure operational and safety conditions. The system includes a warehouse racking structure with 9 storage compartments (3) and 1 dedicated area (4) for dispensing /retrieving products, along with a supporting frame system (1) to facilitate the transfer mechanism (2) to the required locations. The smart sorting section is constructed as shown in Fig. 6.

The parts used to construct the system's mechanical structure are listed in the 3D design file. Due to the scarcity and high cost of some parts, it is custom-designed and utilized 3D printing technology for the manufacturing of complex, intricately structured, and high-precision components to suit the system's structure.

#### 5.1.3 Step-by-step mechanical assembly procedure

**Fig. 7:**
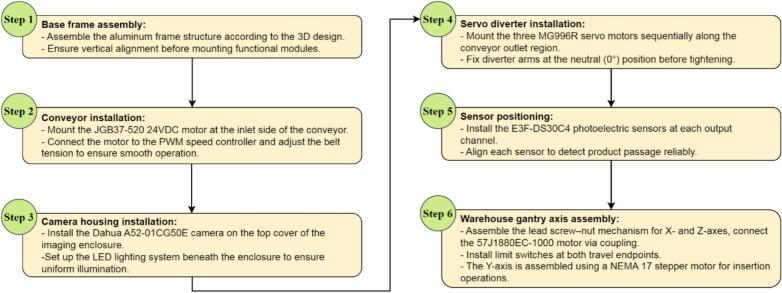
Mechanical assembly sequence of the smart sorting section.

To improve reproducibility, the overall mechanical assembly sequence of the smart sorting section is illustrated in Fig. 7.

Step 1 – Base frame assembly: Assemble the aluminum frame structure according to the 3D design shown in Fig. 5. Ensure vertical alignment before mounting functional modules.

Step 2 – Conveyor installation: Mount the JGB37-520 24VDC motor at the inlet side of the conveyor. Connect the motor to the PWM speed controller and adjust belt tension to ensure smooth operation.

Step 3 – Camera housing installation: Install the Dahua A52-01CG50E camera on the top cover of the imaging enclosure. Position the LED lighting system beneath the enclosure to ensure uniform illumination.

Step 4 – Servo diverter installation: Mount the three MG996R servo motors sequentially along the conveyor outlet region. Fix diverter arms at the neutral (0°) position before tightening.

Step 5 – Sensor positioning: Install the E3F-DS30C4 photoelectric sensors at each output channel. Align each sensor to detect product passage reliably.

Step 6 – Warehouse gantry axis assembly: Assemble the lead screw–nut mechanism for X- and Z-axes, connect the 57J1880EC-1000 motor via coupling, and install limit switches at both travel endpoints. The Y-axis is assembled using a NEMA 17 stepper motor for insertion operations.

**Fig. 8:**
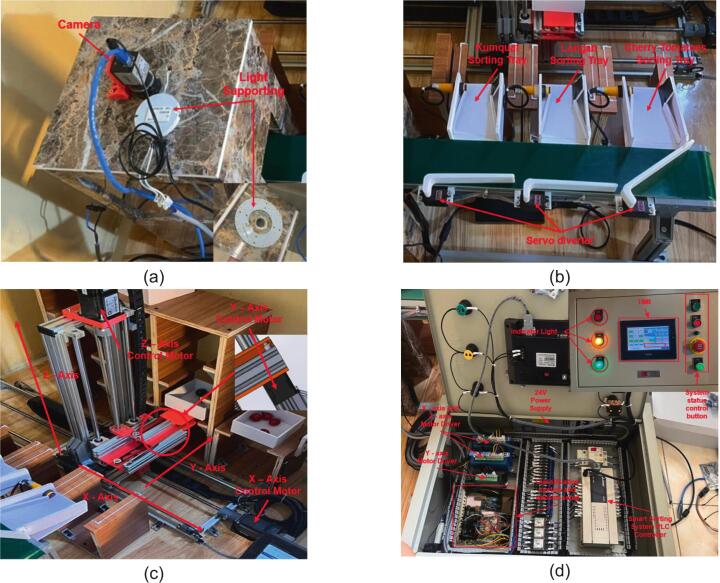
Photographs of the main mechanical modules of the proposed prototype system: (a) Conveyor and vision module, (b) Servo-based fruit diverter mechanism, (c) Warehouse gantry axes (X-Y-Z), (d) Operation control panel.

Fig. 8 presents photographs of the main mechanical modules of the proposed prototype system. The system includes the conveyor and vision module for fruit detection, the servo-based diverter mechanism for sorting, the warehouse gantry axes responsible for storage positioning, and the operation control panel used for monitoring and manual control.

### 5.2 Electrical and control construction

#### 5.2.1 Classification system

**Fig. 9:**
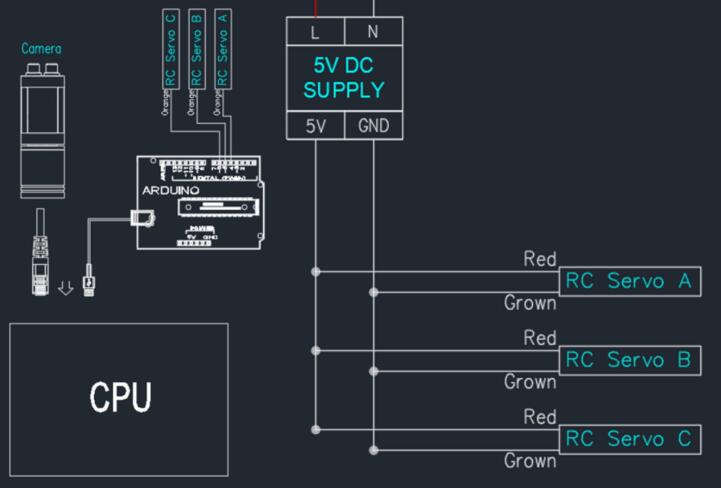
Classification system’s connection diagram.

In the classification system, image acquisition begins with the camera. To be more detailed, the camera has two main connection ports: the first port supplies electrical power for the camera, and the second port is an Ethernet port used to communicate with the computer. Via the second port, the computer conducts signal transmission and image processing with the camera. The connection diagram of the Classification system is described in Fig. 9.

After completing the image processing stage, the host computer transmits the detection results via a serial COM port to the Arduino Uno. The Arduino Uno receives the classification label generated by the YOLOv8 model and forwards the corresponding command to the Arduino Mega. Two microcontrollers are used in the system to separate communication and actuator control tasks. The Arduino Uno is responsible for receiving detection results from the host computer, while the Arduino Mega handles actuator control and communication with the PLC and ESP32 modules. Based on the received classification command, the Arduino Mega actuates the corresponding servo motor to perform the sorting operation. Each servo motor has three wires: two wires for power supply (red for 5V and brown for GND) and one orange wire for signal transmission to the Arduino. Servos A, B, and C are connected to digital control pins 2, 3, and 4 of the Arduino Mega, respectively. The connection diagram of the classification system is illustrated in Fig. 9.

#### 5.2.2 Smart sorting system

**Fig. 10:**
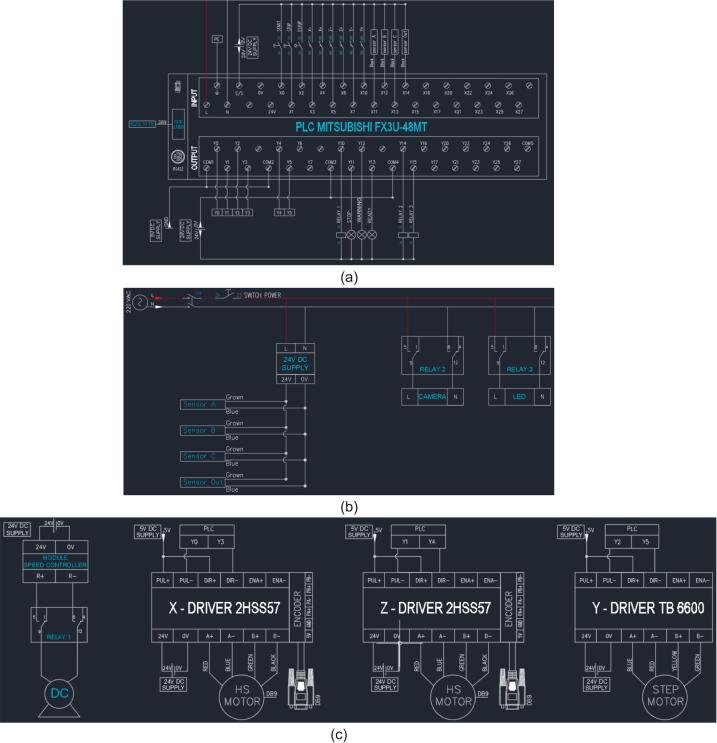
Smart sorting system diagram: (a) PLC connection, (b) Peripheral device connection, (c) Motor connection.

The warehouse control system operates independently of a server and is executed automatically by a Programmable Logic Controller (PLC). The connection terminals, including the input sections and the output sections, are clearly illustrated in Fig. 10. Similar to the classification system, the external peripheral devices of the warehouse system also utilize an external power source to ensure stability.

The connection terminals for the input section on the PLC, are detailed as follows:

- The Start, Stop, and Emergency (E-Stop) push buttons are connected to the terminals from X0 to X2 for overall operation management.
- To detect the rotation direction of the motors, the PLC takes up the input signals on the terminals X3 to X8 for all axes. To be more specific, X3 and X4 are assigned to the X-axis, X5 and X6 to the Z-axis, and X7 and X8 to the Y-axis.
- Three sensors, A, B, and C, connect to input terminals X11–X13 on the PLC to count the quantity of classified products.
- The Out sensor, utilized for monitoring the status of output products, is linked to terminal X14 on the PLC.
- The terminal S/S is used to connected with the 24V power supply to ensure stable operation.

The connection terminals for the output section on the PLC, are detailed as follows:

- The X-Driver of the HS motor is connected to the PLC by using the terminals Y0 and Y3.
- The Y-Driver of the Step motor is connected to the PLC by using the terminals Y2 and Y5.
- The Z-Driver of the HS motor is connected to the PLC by using the terminals Y1 and Y4.
- To ensure the safety of the smart sorting system, the relay network is connected with the PLC via Relay 1, Relay 2, and Relay 3. Particularly, Relay 1 is linked to the terminal Y10 to control the conveyor motor, Relay 2 is linked to the terminal Y14 to control the power supply for the camera, and Relay 3 is linked to the terminal Y15 to control the power supply for the lighting system.
- The light indicators, including STOP, WARNING, and READY are connected to the PLC via the terminals Y11, Y12, and Y13, respectively.
- All PLC output signals are configured using the sink (NPN) wiring method.

#### 5.2.3 Interface system

**Fig. 11:**
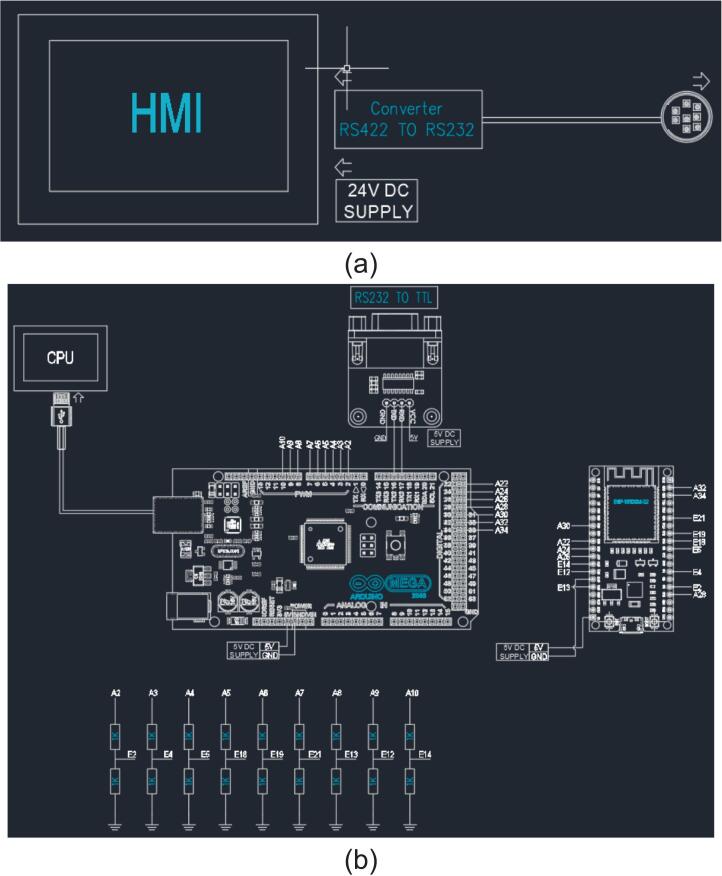
Interface system’s connection diagram: (a) HMI, (b) Digital I/O wiring between Arduino Mega and ESP32 for product count transmission.

The interface system is composed of the Human-Machine Interface (HMI) (a) and the website (b). The HMI is connected to the PLC via an RS422 to RS232 converter to monitor and execute commands with respect to the PLC. The custom – built website component collects data directly through the ESP32 and Arduino MEGA. All information regarding the system's import/export quantities is transferred from the PLC through an RS232 to TTL converter to the control unit, which then pushes the parameters to the pre-built website. The device connections are wired as shown in Fig. 11.

In the system, the ESP32 functions as a data gateway, responsible for collecting data from the Arduino Mega and transmitting it to the View Backend for display on the website. The processing of sensor signals and overall system control logic are handled by the PLC and the Arduino Mega. The Arduino Mega uses the Melsec library to communicate with the PLC by reading data registers. The count values (0 – 3) are encoded in binary form and transmitted through Digital I/O pins using the Parallel Binary Encoding method. The ESP32 directly reads these logic levels to determine the number of products corresponding to each sample type and then updates the data to the web-based system accordingly.

The structure for building the HMI interface and the database for constructing the website are detailed in the system's file design folder (including HMI.txp for the HMI screen interface and web.zip for the website structure).

#### 5.2.4 System calibration and verification procedure

**Servo calibration:** Initialize servo position to 0° and manually align diverter arms before operation.

**Conveyor speed adjustment:** Tune the PWM controller to ensure stable belt motion without vibration or slipping.

**Sensor sensitivity adjustment:** Adjust the E3F-DS30C4 sensor sensitivity knob to ensure stable switching during product passage.

**Limit switch verification:** Manually move each axis to confirm proper limit switch activation and motion interruption.

### 5.3 Embedded and PLC control algorithms

To complement the AI-based detection description, this section provides an explicit algorithmic description of the embedded control logic (Arduino Mega) and the PLC-based warehouse control logic. The control architecture is divided into two layers: (1) the embedded classification controller and (2) the PLC-based warehouse controller.

#### 5.3.1 Embedded classification control logic (Arduino Mega)

The classification subsystem is governed by a state-based control algorithm implemented on the Arduino Mega. The objective of this algorithm is to ensure deterministic sequential actuation of the servo motors while preventing mechanical overlap.

The embedded control logic follows a finite-state machine (FSM) sequence:

**State S0 – Initialization (Idle)**

All servo motors are set to the neutral position (0°). The system waits for the classification result from the host computer.

**State S1 – Label reception**

Upon receiving a classification label (A, B, or C), the Arduino selects the corresponding servo motor. If no valid label is received, the system remains in Idle state.

**State S2 – Actuation**

The selected servo rotates to a predefined sorting angle to redirect the product into the designated output channel.

**State S3 – Sensor confirmation and counting**

When the photoelectric sensor detects product passage, the count value is incremented by one and transmitted to the PLC.

**State S4 – Reset**

The servo returns to its neutral position before processing the next product. The system processes one product at a time to avoid simultaneous actuation or mechanical interference.

#### 5.3.2 PLC warehouse control logic

The PLC control logic can be represented as the following sequential operation algorithm. The PLC program operates under the cyclic scan model typical of industrial PLC systems. Each scan cycle consists of input reading, logic execution, and output update.

During each scan cycle, the PLC reads sensor inputs (X11–X14), mode selection (Auto/Manual), and safety signals. Real-time product counts are compared with preset thresholds configured via the HMI interface.

In Auto mode, when a preset condition is satisfied, the PLC executes a predefined axis movement sequence:

(1) Move X-axis to the target coordinate;
(2) Move Z-axis to the required vertical position;
(3) Activate Y-axis insertion mechanism;
(4) Return axes to standby position.

In Manual mode, the PLC moves axes to user-selected coordinates via the HMI command.

Safety interlocks ensure that limit switch activation immediately stops the corresponding axis, and Emergency Stop disables all PLC outputs. After completing each scan cycle, the PLC returns to the input scanning stage and repeats the process.

## 6 Operation instructions

### 6.1 System setup and firmware installation procedure

To ensure full reproducibility of the proposed open-hardware system, the complete software setup, firmware installation, communication configuration, startup sequence, and subsystem testing procedures are described in detail below.

To further facilitate reproducibility, demonstration videos illustrating the mechanical assembly process and the practical system setup procedure are provided through the project repository. The repository includes links to publicly accessible videos that demonstrate the assembly simulation of the system modules and the real-world setup and configuration procedure of the prototype system.

#### 6.1.1 Required software environment

The following software tools are required to configure and operate the system:

– Python 3.10 or later for running the YOLOv8 detection model.
– Ultralytics YOLOv8 library installed via pip install ultralytics.
– Arduino IDE (version 2.x or later) for uploading firmware to the Arduino Mega and ESP32.
– Mitsubishi GX Works2 for opening and downloading the PLC.gxw project file to the FX3U-48MT/ES PLC.
– TouchWin V1.1 for editing and downloading the HMI.txp project file to the TG765S-XT HMI screen.

The PLC control program for the Mitsubishi FX3U-48MT/ES is developed and edited using Mitsubishi GX Works2 (version 1.560J or later). The PLC.gxw project file must be opened using GX Works2, and the program is downloaded to the FX3U-48MT/ES PLC via the standard Mitsubishi programming interface cable before switching the PLC to RUN mode.

#### 6.1.2 Firmware flashing and configuration sequence

Step 1 – Upload Code_mega.ino to Arduino Mega (board: Arduino Mega 2560; baud rate: 9600 bps).

Step 2 – Open PLC.gxw using GX Works2 and download the program to the FX3U-48MT/ES PLC; then switch PLC to RUN mode.

Step 3 – Upload ESP32_code.ino to the ESP32 board and configure WiFi credentials.

Step 4 – Open HMI.txp in TouchWin V1.1, configure communication parameters, and download to the Xinje TG765S-XT HMI.

#### 6.1.3 Communication configuration parameters

- Arduino–PLC communication is implemented via the Melsec library over RS232

- Product count values are transmitted from Arduino to ESP32 using parallel Digital I/O binary encoding.

- ESP32 transmits count data to Firebase via WiFi TCP/IP.

- HMI communicates with PLC via RS422-to-RS232 interface.

#### 6.1.4 Power-on and startup sequence

1. Supply 24V power to PLC and sensors.
2. Switch PLC to RUN mode.
3. Power on Arduino Mega and ESP32.
4. Start the host computer and execute Detectyolov8.py.
5. Verify camera live stream.
6. Press Power and Start buttons to begin operation.

#### 6.1.5 Subsystem testing before automatic operation

- Conveyor verification via PWM speed tuning.
- Servo actuation verification and reset confirmation.
- Sensor triggering and count increment validation.
- Warehouse axis movement verification using HMI JOG mode.
- HMI display confirmation for coordinates, counts, and system status indicators.

### 6.2 Data Preprocessing

**Fig. 12:**
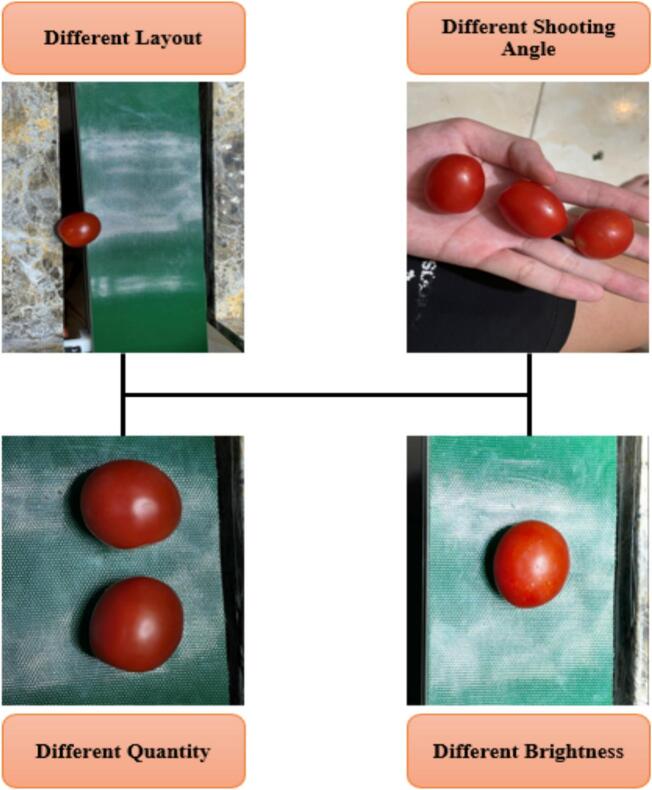
Diversification of image samples.

To start with, the raw image dataset is obtained on three typical fruits in Viet Nam, including kumquats, longans, and cherry tomatoes. Moreover, several data augmentation techniques are applied to diversify the dataset as well as create a more robust and comprehensive one. These algorithms are conducted in two directions: the sample positions and the color characteristics. To be more detailed, the algorithms related to sample positions concentrate on changing the arrangement of fruits within the image region, changing the quantity of fruits in each image, and capturing images from different shooting angles. Meanwhile, the algorithms related to color characteristics concentrate on changing the lighting conditions, modifying color saturation, changing to gray images, and so on. Based on the new dataset, the performance of the model training process could be better across different scenarios encountered in real-world applications. Fig. 12 indicates some results of the image augmentation process by aiding different techniques.

Additionally, publicly available datasets were incorporated to increase the diversity of the collected samples. The initial dataset collected from laboratory acquisition and public datasets contains 537 raw images. Each image may include multiple fruit instances belonging to three categories: longan, kumquat, and cherry tomato. The annotation process was conducted using the Roboflow platform, where each fruit instance was manually labeled with bounding boxes corresponding to the three categories. To improve model robustness, several data augmentation techniques were applied, including image resizing, rotation, flipping, and brightness adjustments. After applying these augmentation techniques, the dataset was expanded to 1456 images. The final dataset consists of 1379 images for training and 77 images for validation. This split ratio was selected to maximize the number of training samples given the relatively small dataset size while still preserving a validation set for monitoring training convergence. The validation set size is relatively small due to the limited dataset size; however, it was sufficient to monitor training convergence and prevent overfitting during the training process. All augmentation operations were applied only to the training set after the dataset splitting process to avoid data leakage between the training and validation sets.

The image acquisition process is conducted under controlled laboratory conditions. The conveyor system and camera module are installed inside an enclosed chamber equipped with a fixed LED illumination system positioned beneath the camera. The illumination intensity is maintained approximately constant throughout data collection to reduce shadow variation.

The camera is mounted vertically above the conveyor at a fixed height of approximately 25–30 cm, with a perpendicular viewing angle relative to the object surface. The background consists of a uniform conveyor belt surface to minimize visual noise.

To improve dataset diversity, images are captured with slight variations in object orientation and position along the conveyor. Additional brightness and contrast augmentations are applied during preprocessing to improve robustness to illumination variation. Partial occlusion scenarios are introduced by placing overlapping fruit samples to simulate more complex detection cases.

It should be noted that the dataset is collected under controlled indoor lighting conditions; therefore, the trained model is primarily optimized for laboratory environments.

### 6.3 Communication Protocols

**Fig. 13:**
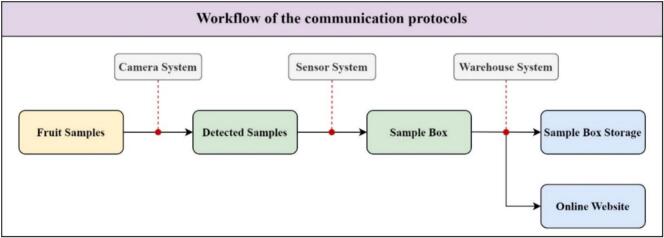
The workflow of the communication protocols.

Fig. 13 shows the workflow of the communication protocols. The samples are firstly placed on the conveyor belt. When the conveyor is passing through the camera system, the image signal of the samples is obtained and then transmitted from the camera to the host computer. Thereafter, the YOLOv8 model on the host computer identifies the fruit type for each sample. This detection result is transmitted to the Arduino microcontroller to generate the suitable commands for the actuators. The sensor system is used to count the number of samples for each kind of fruit. After completing the classification phase, each sample is placed in the correct sample box. The PLC controller publishes the commands for the warehouse system to transport the containers to the designated storage slots once the sample box is full. The sensor system also gathers data on the number of sample containers in the storage during this phase. The primary communication protocol used between the controllers is the standard RS232 protocol. To be more detailed, the Arduino microcontroller and the PLC controller are defined as RS232 to TTL communication converters, whereas the HMI and PLC controller are defined as RS422 to RS232 communication converters. Furthermore, the proposed system operates in two primary modes, referred to as Manual and Auto. This configuration aims to ensure safety among several operational stages for the system as well as address any necessary troubleshooting.

**Fig. 14:**
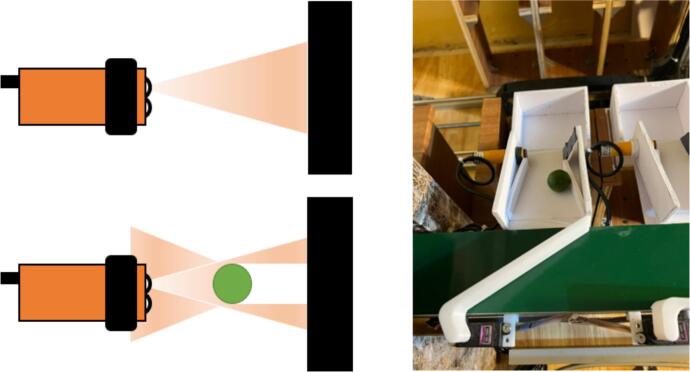
The operating principle of E3F-DS30C4 sensor.

The sensor system is described in Fig. 14. The system uses the E3F-DS30C4 sensors, which consist of an infrared emitter and a reflective signal receiver. In a normal state, the infrared beam is projected onto a black wall surface, where the light is absorbed and prevents any reflected signal from reaching the receiver. When a sample passes through the sensor, the infrared beam is reflected based on the sample's color. The change is then detected by the signal receiver of the sensor. This procedure triggers the output signal from “0” to “1” and sends it to the PLC. Consequently, the number of times the signal triggers matches the counted samples.

All the system operation data, including errors during operation, is displayed on the HMI screen. Moreover, the data about the quantity of the fruit samples and the container are also obtained and stored in real-time. More specifically, we have created an online platform that offers essential information to both sellers and consumers. Based on that, they are able to easily track all items in the smart warehouse.

**Fig. 15:**
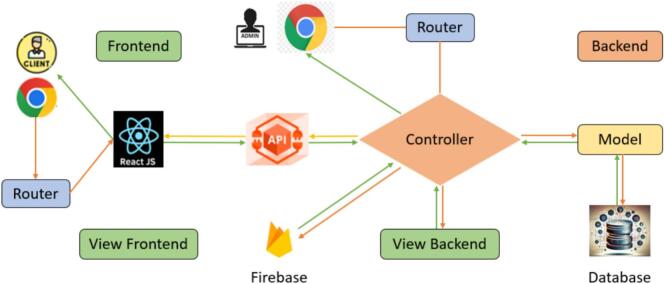
The web project designed for the system.

Fig. 15 indicates the web project designed for the system. Frontend, Backend, Controller, and Model are three primary components of the general architecture of the web system. The function of all components is demonstrated as in Table 5.

**Table 5:** The functions of the components developed for the system web project.

**No.**	**Components**	**Functions**
1	Frontend	Frontend ensures an aesthetically pleasing and user-friendly experience via the layout, colors, and images that present website information to users.
2	View Frontend	View Frontend is developed using React and used for rendering the interface, visual effects, and data to the Client.
3	Controller	The Controller acts as the core of the application that retrieves data through the Model and returns results in the form of APIs or rendered interface content.
4	Backend	Backend manages the website's features, including offers, commodities, orders, the distribution of administration rights, and so on.
5	View Backend	View Backend contains interface-related code which the Controller uses to integrate processed data before sending the final response to the user.
6	Firebase	Firebase serves as a real-time data storage and synchronization intermediary between the ESP32 and the web system. To be more detailed, the ESP32 module transmits sensor data to Firebase. Based on that, the system retrieves and displays the information on the website. In addition, the system only performs monitoring functions and does not implement any mechanism to send control commands back to the ESP32 module.
7	Model	Model represents the system’s data structure and connects to the Database to fetch data upon the Controller’s request.
8	Database	Database stores all data required for system operation and display.

On the embedded side (ESP32), configuration parameters such as API_KEY and DATABASE_URL are defined in the firmware to establish a secure connection to the Firebase Realtime Database. Firebase security rules are configured through the Firebase Console to enable appropriate read and write permissions, ensuring reliable real-time data transmission from the sensor system during product quantity updates.

On the backend side, the system integrates Firebase using the Admin SDK within a Node.js environment. The Controller components in the MVC architecture retrieve data from Firebase and expose it through RESTful APIs. These APIs are subsequently consumed by the frontend via the Axios library for data processing and visualization tasks, including inventory tracking and system status monitoring.

### 6.4 Operation process

**Fig. 16:**
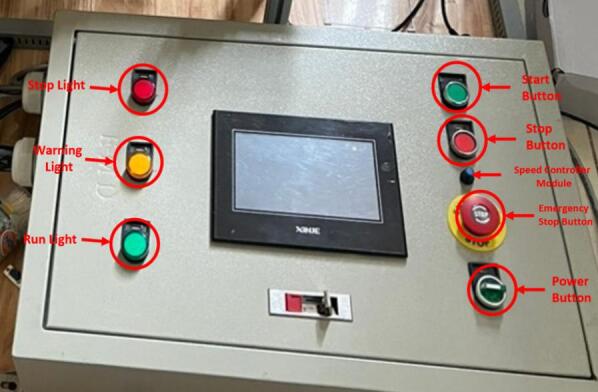
System management interface.

The system management interface is clearly illustrated in Fig. 16. On the left side of the control board, there are three indicator lights that represent the working states of the system: Stop Light, Warning Light, and Run Light. Meanwhile, all the system's operating buttons are located on the right side of the control board. To be more specific, the power source is activated by using the Power button and can be immediately emergency shutdown when the Emergency Stop button is pressed. To start or stop the system operation, the operator presses the Start/Stop button after the power has been supplied, at which point the system begins or ceases operation accordingly. In addition, the conveyor speed can be adjusted using the potentiometer on the motor speed control module.

**Fig. 17:**
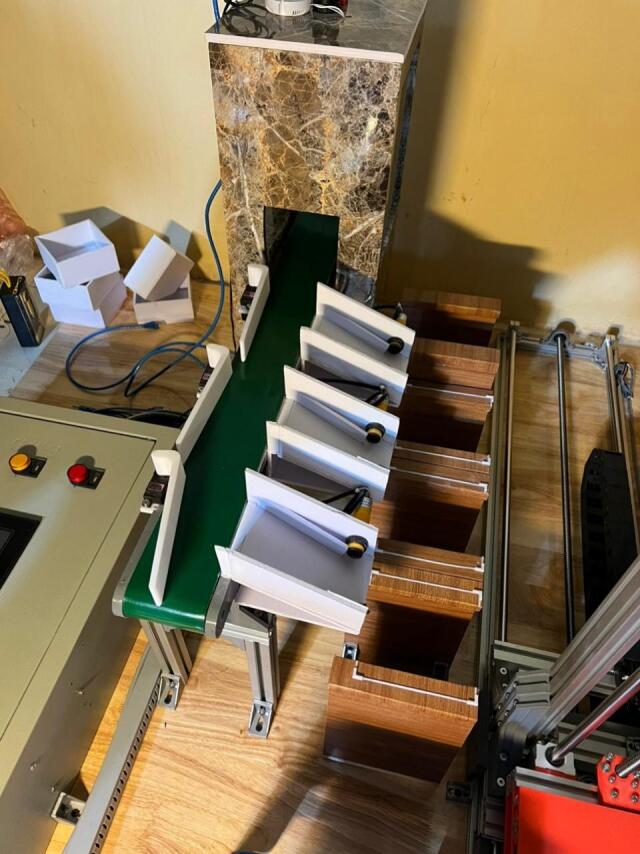
Classification process.

Fig. 17 illustrates the classification process of the system. Once the system is started operating, the motor, camera, and LED lighting are activated. The product is moved from the beginning of the conveyor into the camera housing to perform the image acquisition process. After the server applies the trained AI model to detect the type of each product, it sends the suitable control signals to the Arduino. The Arduino then actuates the corresponding servo motor to rotate the diverter arm, allowing the product to roll down into its designated classified tray. As the product on the output tray is detected by the sensor, the system increments the number of the counted products by one unit. This mechanism is then repeated and applied similarly for the next products. Hence, the proposed system can operate sequentially in real time, which is suitable for industrial environments.

**Fig. 18:**
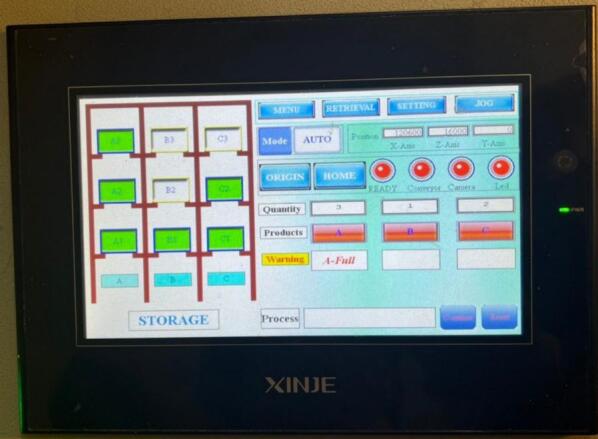
Smart sorting process interface.

Fig. 18 shows the interface of the smart sorting process. It is noticed that once the sensor counts enough products according to the preset standard and displays the notifications on the HMI, the system can select one of two predefined modes (Auto or Manual) for the next step. With the Auto mode, the warehouse system automatically places the storage boxes into correct programming positions for each product type. With the Manual mode, the operator can select a specific position, and the warehouse will move the selected box to that location. The coordinates of each storage slot on the rack have been predefined according to the physical layout of the shelving system. Similarly, for the outbound process, the operator can select a specific storage slot, and then the system retrieves and transports the box to the dispatch position. Additionally, the HMI interface always displays the real-time coordinates of each axis. This allows the operator to use the JOG function to recalibrate and fine-tune the entire system if necessary.

## 7 Validation and characterization

### 7.1 Training results on different models

**Fig. 19:**
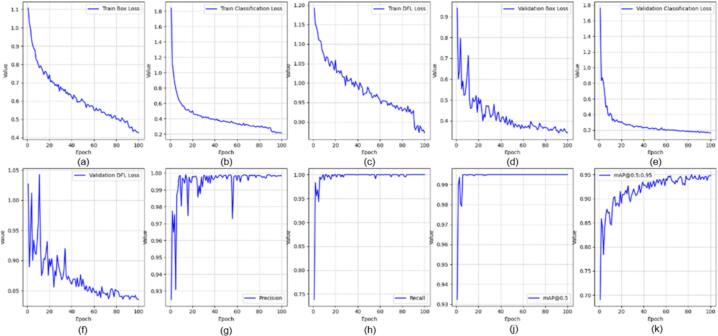
Training and validation results of the YOLOv8-MIF model: (a) Train Box Loss, (b) Train Classification Loss, (c) Train DFL Loss, (d) Validation Box Loss, (e) Validation Classification Loss, (f) Validation DFL Loss, (g) Precision, (h) Recall, (j) mAP @0.5, (k) mAP @0.5:0.95.

**Fig. 20:**
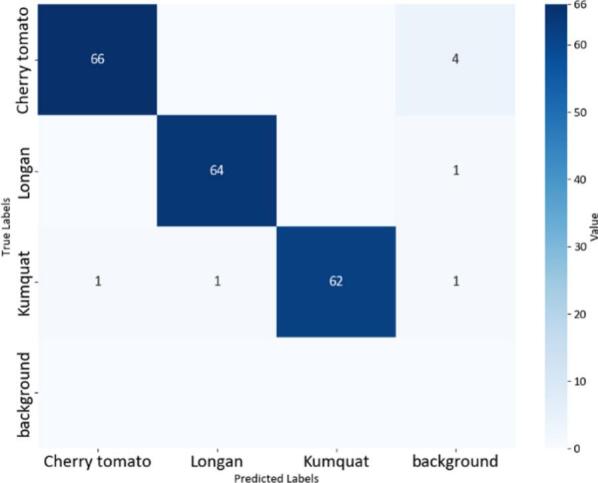
Confusion matrix of the YOLOv8-MIF model.

**Fig. 21:**
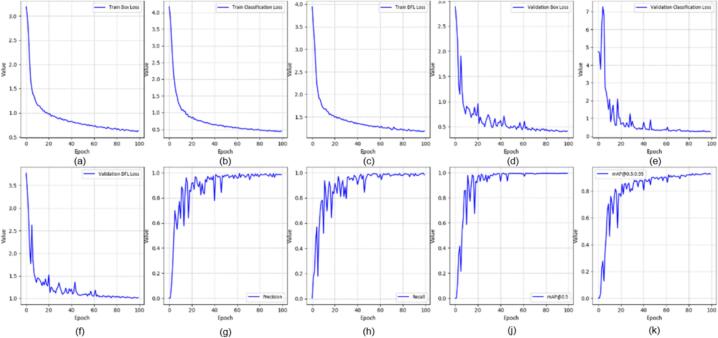
Training and validation results of the YOLOv8n model: (a) Train Box Loss, (b) Train Classification Loss, (c) Train DFL Loss, (d) Validation Box Loss, (e) Validation Classification Loss, (f) Validation DFL Loss, (g) Precision, (h) Recall, (j) mAP @0.5, (k) mAP @0.5:0.95.

All YOLOv8 models were trained using the Ultralytics framework for 100 epochs with an input resolution of 640 × 640 pixels and a batch size of 16. The initial learning rate was set to 0.001 using the Adam optimizer. In this section, we evaluated several YOLOv8-based variants for real-time application, including YOLOv8-MIF [20], YOLOv8n [21], YOLOv8+GAM Attention [22], YOLOv8-CBAM, and YOLOv8m+ECA [23]. Fig. 19, Fig. 20, Fig. 21 depict the effectiveness of YOLOv8 models on the data set. Fig. 19 shows the training results of the YOLOv8-MIF model. The model converges well, with accuracy increasing steadily across epochs. The loss value gradually decreases over time, indicating the model's reliability. However, this model still has fluctuations in the middle stage of training. Fig. 21 presents the training process of YOLOv8n, a lightweight version in the YOLOv8 family. The graph shows that accuracy increases rapidly and stabilizes early. The loss decreases steadily and shows no significant fluctuations. Fig. 23 illustrates the results using the YOLOv8-GAM Attention model. The accuracy curve increases sharply from the first epochs. The loss decreases rapidly and remains stable. This reflects the positive impact of the model in extracting important features from the dataset. In Fig. 25, the YOLOv8-CBAM model shows slight loss fluctuations at the beginning. However, it quickly reaches a stable state in the middle and late stages of training. Finally, Fig. 27 presents the training process of the YOLOv8m-ECA model, which shows the best performance among all. The loss decreases very quickly and consistently while accuracy increases sharply and reaches a near-optimal level after only a few epochs.

**Fig. 22:**
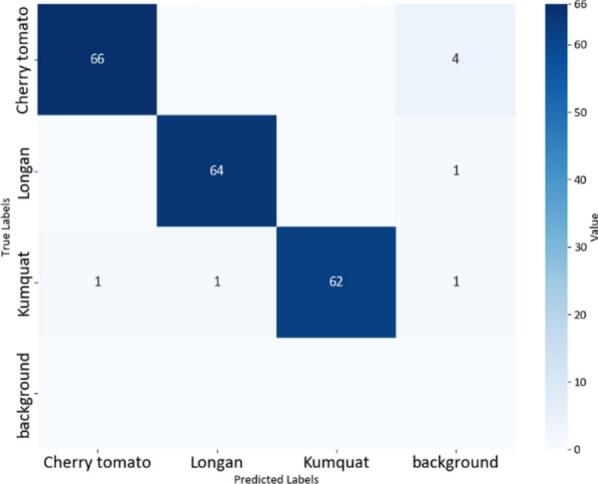
Confusion matrix of the YOLOv8n model.

**Fig. 23:**
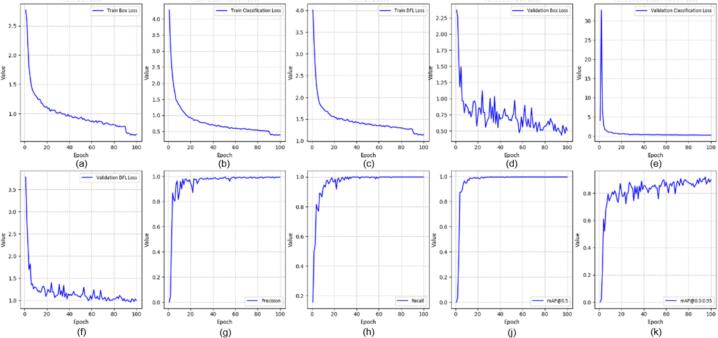
Training and validation results of the YOLOv8-GAM Attention model: (a) Train Box Loss, (b) Train Classification Loss, (c) Train DFL Loss, (d) Validation Box Loss, (e) Validation Classification Loss, (f) Validation DFL Loss, (g) Precision, (h) Recall, (j) mAP @0.5, (k) mAP @0.5:0.95.

**Fig. 24:**
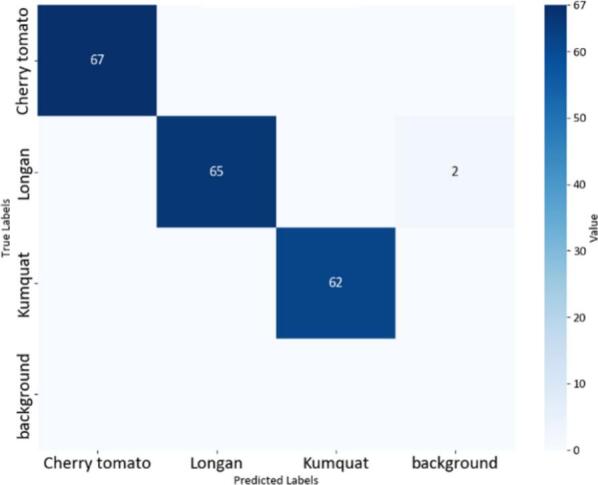
Confusion matrix of the YOLOv8-GAM Attention model.

The confusion matrices in Fig. 20, Fig. 21, Fig. 22 provide a clear and measurable view of how well the YOLOv8 models classify the fruit images. In Fig. 20, the YOLOv8-MIF model correctly classifies most of the samples, with high values appearing along the main diagonal. However, there are still a few small mistakes between some classes, especially those that look similar in color or shape. Fig. 22 shows the performance of the YOLOv8n model, which is a lighter version. It also performs well in separating the classes. In Fig. 24, the YOLOv8-GAM Attention model gives almost perfect results. The diagonal values are very high, and the values outside the diagonal are nearly zero, meaning there are almost no misclassifications. Fig. 26 shows the results of the YOLOv8-CBAM model, which classifies most samples correctly. However, compared to models that use attention mechanisms, there are still a few wrong predictions outside the diagonal. Lastly, Fig. 28 presents the confusion matrix of the YOLOv8m-ECA model, which performs the best among all. Almost all samples are classified correctly, with values focused along the diagonal and no noticeable errors.

**Fig. 25:**
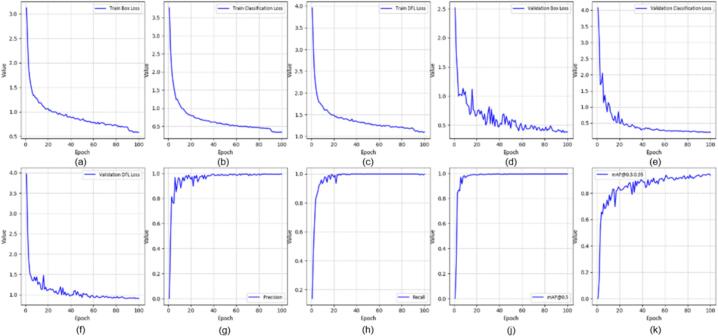
Training and validation results of the YOLOv8-CBAM model: (a) Train Box Loss, (b) Train Classification Loss, (c) Train DFL Loss, (d) Validation Box Loss, (e) Validation Classification Loss, (f) Validation DFL Loss, (g) Precision, (h) Recall, (j) mAP @0.5, (k) mAP @0.5:0.95.

**Fig. 26:**
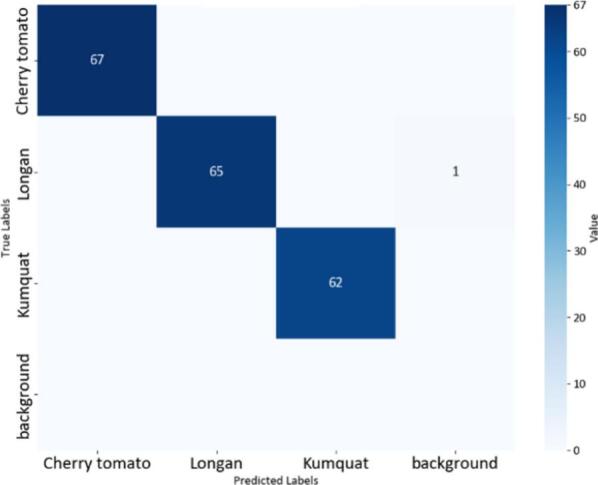
Confusion matrix of the YOLOv8-CBAM model.

**Fig. 27:**
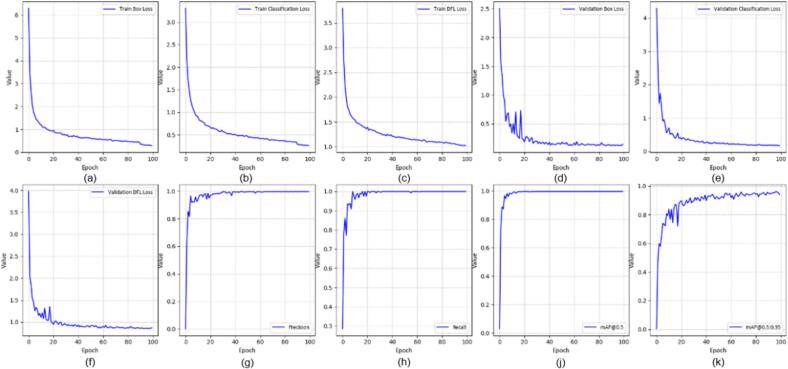
Training and validation results of the YOLOv8m-ECA model: (a) Train Box Loss, (b) Train Classification Loss, (c) Train DFL Loss, (d) Validation Box Loss, (e) Validation Classification Loss, (f) Validation DFL Loss, (g) Precision, (h) Recall, (j) mAP @0.5, (k) mAP @0.5:0.95.

**Fig. 28:**
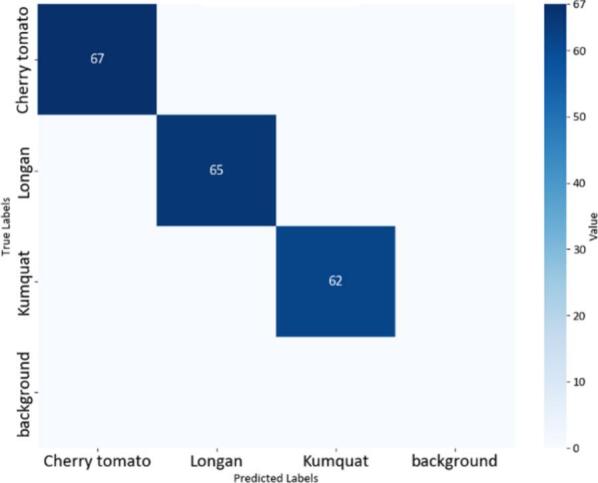
Confusion matrix of the YOLOv8m-ECA model.

Table 6 shows the performance of different YOLOv8 model variants using five key metrics including Accuracy, F1-Score, Precision, Recall, and Validation DFL Loss. The YOLOv8m+ECA model gives the best results. It reaches an accuracy of 99.99% with an F1-score approaching 1. The YOLOv8-CBAM has an accuracy of about 99.48%, and the F1-score is approximately 1. However, its validation loss is a bit higher (0.90793) than that of YOLOv8m+ECA. The YOLOv8+GAM Attention model has a precision of 0.98682 and a validation DFL loss of 0.95798. The YOLOv8n model performs well too. It reaches a high precision (0.99727) and has a low validation loss (0.84029). Among all evaluated models, YOLOv8-MIF shows the lowest performance with the lowest accuracy and the highest validation DFL loss. Its accuracy is only 96%, and it has the highest validation DFL loss (1.0105). It should be noted that the high accuracy values obtained in this study are partly attributed to the controlled laboratory conditions and the limited number of fruit categories.

**Table 6:** Comparison results among different versions of YOLOv8 models.

	**Accuracy**	**F1-Score**	**Precision**	**Recall**	**Validation DFL Loss**
**YOLOv8-MIF**	96.00%	0.99	0.99727	0.9954	1.0105
**YOLOv8n**	98.97%	1	0.99727	0.9954	0.84029
**YOLOv8+GAM Attention**	98.97%	1	0.98682	1	0.95798
**YOLOv8+CBAM**	99.48%	1	0.99523	0.99323	0.90793
**YOLOv8m+ECA**	**99.99%**	1	**0.99709**	1	**0.84735**

### 7.2 Evaluate the system functions

#### 7.2.1 The recognition ability

**Fig. 29:**
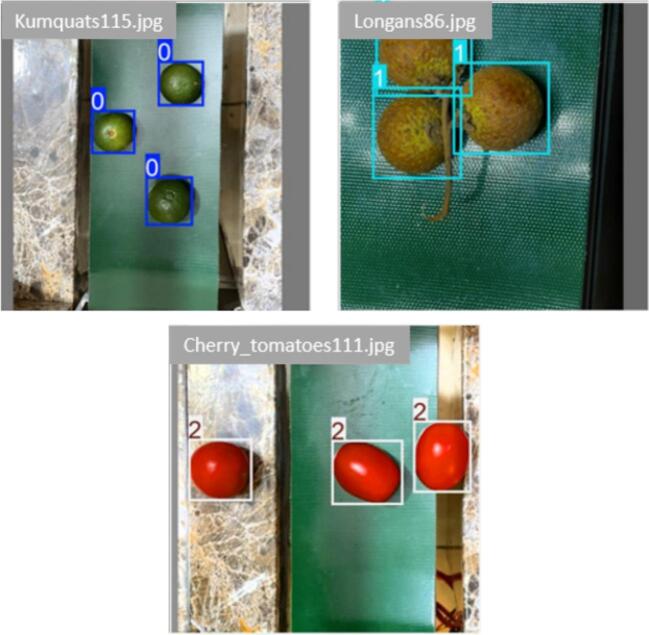
Real-time fruit detection results of the proposed system.

As shown in Fig. 29, three varieties of fruits are detected and classified within the image frame. With particular number labels applied to each type of fruit, they are labelled with a specific code number: kumquats as “0,” longans as “1,” and cherry tomatoes as “2”. Each fruit sample is enclosed by a color bounding box. It is evident that even though the fruit samples are set up at different coordinates or placed in several directions in the image region, the proposed model successfully detects all fruit instances. Furthermore, in certain instances where samples overlap, the proposed model accurately recognizes each one.

#### 7.2.2 The classification ability

The detected signals are sent to the central controller, which handles and analyzes the data to ascertain the proper categorization for every item after its passage over the classification camera system. The deep learning model, YOLOv8, precisely identifies the fruits in real-time, driving this system. After that, the controller transmits control signals to the sorting system, whose actuators guide the products into the proper storage spaces.

**Fig. 30:**
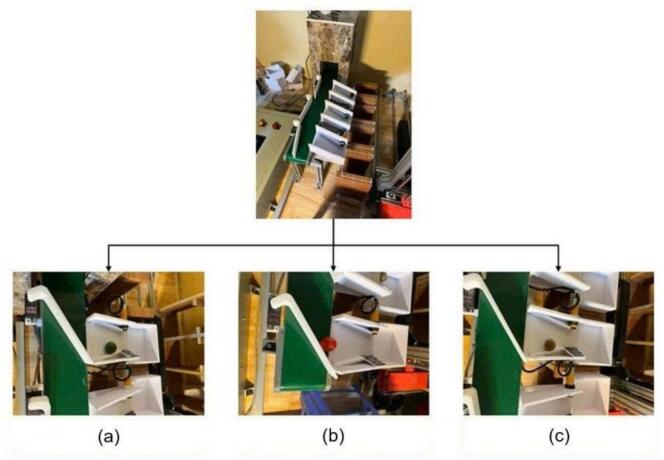
The operation of the classification system: (a) kumquats classification, (b) cherry tomatoes classification, (c) longans classification

The sorting mechanism navigates the objects automatically to one of three storage sections: the first for kumquats (a), the second for longans (b), and the third for cherry tomatoes (c). This sorting system improves automation efficiency and minimizes the need for human labor. Fig. 30 shows the exact distribution of every fruit into its corresponding compartment; therefore, it illustrates the sorting and classification process. Apart from simplifying the sorting process, this system guarantees high accuracy and efficiency in controlling the smart warehouse, thereby maximizing space use and enhancing inventory management.

#### 7.2.3 The smart sorting ability

**Fig. 31:**
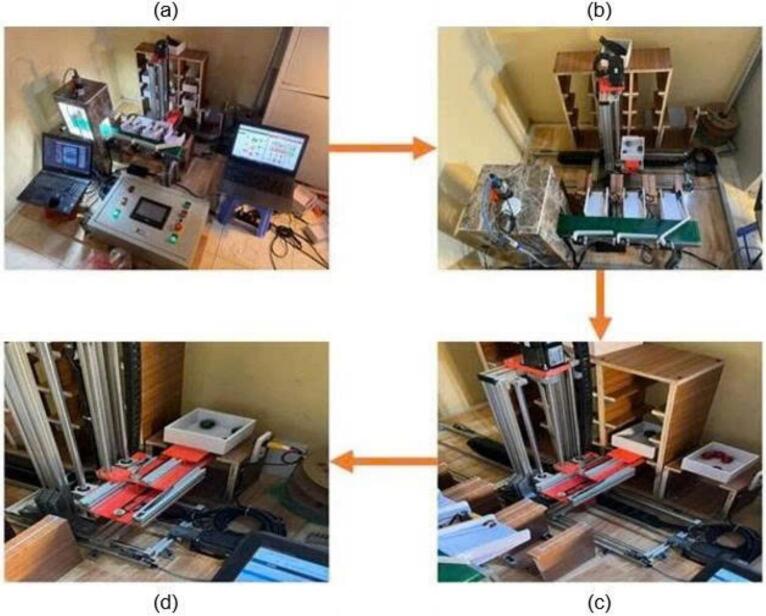
The operation of the warehouse system: (a) standby position, (b) picking position, (c) storing position, (d) delivering position

As illustrated in Fig. 31, the automated warehousing system executes a precise sequence of tasks to ensure the smooth and efficient processing of products. The robotic arm is triggered to move objects to their intended storage places after the controller gets data from the sensors on the item count. These locations are preprogrammed and automatically selected. They are adaptable in case human adjustments are required. The system goes into a standby condition. It becomes ready to start operations once the products are delivered successfully. This orderly flow maintains the performance and reliability of the system in inventory management. It offers amazing efficiency and accuracy in storage, retrieval, and distribution.

#### 7.2.4 Evaluate the system operation speed in real–time

During experimental operation, the complete system achieved an overall classification accuracy of 98% (196 correct classifications out of 200 trials). While the YOLOv8m+ECA model achieved a detection accuracy of 99.99% on the dataset, the overall system-level classification accuracy measured during real-time operation was approximately 98%. The average processing speed is approximately 20 products per minute. The system has been experimentally tested to operate continuously for up to 24 hours without mechanical or control failure, demonstrating stable operation under laboratory conditions. Although there are some operational limitations (such as recognition latency, limited robustness to harsh environments, and dependence on transmission stability), the experimental results indicate that the system operates stably under laboratory conditions.

### 7.3 Power consumption analysis

To enhance the engineering completeness and industrial relevance of the proposed system, an estimation of power consumption and energy usage is provided.

The power consumption of the system is calculated based on the rated specifications of individual components. The main subsystems and their approximate rated power are summarized as follows:

– Conveyor DC motor (24V): approximately 60 W

– Three MG996R servo motors (peak combined load): approximately 45 W

– Two stepper motors (57J1880EC-1000) with 2HSS57 drivers: approximately 220 - 320 W

– Nema17 + driver TB6600: approximately 48 - 58 W
– Mitsubishi FX3U-48MT/ES PLC: approximately 25 W
– HMI Xinje TG765S-XT: approximately 15 W
– Camera Dahua A52-01CG50E: approximately 8 W
– Arduino Mega controller: approximately 5 W
– ESP32 module: approximately 3 W
– LED illumination system: approximately 20 W
– Host computer running YOLOv8 inference: approximately 200 - 220 W

Under nominal operating conditions, the estimated total rated power of the complete system is approximately 0.65 – 0.8 kW, calculated from the rated specifications of the individual components.

The average duration of one classification cycle (including image acquisition, inference, mechanical actuation, and counting) is approximately 3 – 4 seconds. The energy consumption per classification cycle can therefore be estimated using:

(6) $$E=P_{\mathrm{avg}}\times t_{\mathrm{cycle}}$$

where: $P_{\mathrm{avg}}$ is the average system power during operation and $t_{\mathrm{cycle}}$ is the time required per sorting cycle.

Based on the nominal power range, the estimated energy consumption per classification cycle is approximately 0.55–0.90 Wh.

It should be noted that these values are derived from rated component specifications rather than long-term industrial measurements, as the system is validated at laboratory scale for functional and integration verification. Actual power consumption may vary depending on duty cycle, load conditions, and environment.

Compared to manual sorting operations, which require continuous human labor and sustained lighting conditions, the proposed system provides predictable and stable electrical consumption while significantly reducing labor intensity. Although quantitative industrial-scale benchmarking was beyond the scope of this prototype study, the laboratory-scale energy estimation demonstrates the feasibility of integrating the system into smart warehouse environments with controlled and manageable energy demand.

Compared with existing studies in automated fruit classification, the proposed system demonstrates competitive classification accuracy and a higher level of system integration. For instance, YOLOv5-based approaches such as Yazid et al. reported an accuracy of approximately 96%, whereas the proposed YOLOv8m+ECA model achieves up to 99.99% accuracy in our experimental evaluation.

In contrast to approaches that primarily focus on robotic manipulation (e.g., Gaspar et al.), this study implements a fully integrated data pipeline, where image data captured by a Dahua camera are processed by the YOLOv8-based model, synchronized in real time via Firebase and ESP32, and subsequently used to control a PLC-based warehouse management system.

With a processing speed of approximately 20 products per minute and a stable energy consumption ranging from 0.55 to 0.90 Wh per cycle, the system improves operational efficiency compared to manual processes while providing a scalable smart warehouse solution with web-based monitoring and control.

### 7.4 Conclusions

The paper proposes a smart automation system integrated with computer vision algorithms and a deep learning model to classify fruit. To be more specific, the YOLOv8 network is trained on the constructed fruit image dataset to recognize three types of common Vietnamese fruit: kumquats, longans, and cherry tomatoes. The Arduino microcontroller receives the detection results and sends the commands for classifying the fruits into their respective storage boxes. After that, the fruit-filled boxes are placed into the desired storage slots in the smart warehouse system by using the programming logic controller. The obtained information about the sample quantity and classification results during the entry-exit process is updated continuously on the online website. Through the model training results and different real-time inspection cases, the efficiency and accuracy of the system demonstrate promising performance. These results demonstrate that the proposed system has strong potential for practical deployment in automated production and smart warehouse environments.

Although the experimental validation in this study was conducted using three fruit categories, the proposed system architecture is inherently modular and scalable.

From the AI perspective, the YOLOv8-based detection framework natively supports multi-class object detection. Extending the system to additional fruit types requires collecting new labeled datasets and retraining the model with expanded class definitions; no structural modification of the neural network architecture is required.

From the hardware perspective, the classification module can be scaled by adding additional servo actuators and corresponding output channels. However, for a large number of categories, linear servo-based diversion may become inefficient. In such cases, alternative scalable mechanisms such as branched conveyor systems or rotary sorting wheels can be implemented to improve throughput and classification capacity.

The warehouse subsystem is coordinate-based and parameterized. Expanding the number of fruit categories does not require fundamental changes to the PLC program structure; instead, the mechanical travel range of the axes can be extended and additional storage racks can be integrated. Storage coordinates can then be updated via the HMI configuration interface.

Additionally, the current implementation uses parallel Digital I/O binary encoding between Arduino and ESP32 for count transmission (e.g., 0–3 encoded using two I/O pins). While suitable for limited category counts, larger-scale implementations would benefit from adopting serial communication protocols (e.g., UART or I2C) to reduce I/O consumption and improve scalability. All mechanical design files, control firmware, and datasets are publicly available to ensure reproducibility and facilitate further research and development by the community.

**Ethics statements**

This study did not involve any human subjects and animal experiments

## CRediT authorship contribution statement

**Thi-Thoa Mac:** Conceptualization, Methodology, Resources, Supervision, and Project administration.. **Huy-Anh Bui:** Writing – review & editing, Software, Formal analysis, Data curation. **Duc-Vinh Pham:** Writing – original draft, Visualization, Software, Formal analysis, Data curation. **Hoang-Hiep Ly:** Writing – original draft, Visualization, Validation, Resources, Methodology, Formal analysis, Data curation. **Xuan-Thuan Nguyen:** Writing – review & editing, Writing – original draft, Validation, Supervision, Project administration, Methodology, Data curation, Conceptualization.

## Declaration of competing interest

The authors declare that they have no known competing financial interests or personal relationships that could have appeared to influence the work reported in this paper.
